# Study of 11 BMI-Associated Loci Identified in GWAS for Associations with Central Obesity in the Chinese Children

**DOI:** 10.1371/journal.pone.0056472

**Published:** 2013-02-12

**Authors:** Bo Xi, Hong Cheng, Yue Shen, Giriraj R. Chandak, Xiaoyuan Zhao, Dongqing Hou, Lijun Wu, Xingyu Wang, Jie Mi

**Affiliations:** 1 Department of Maternal and Child Health Care, School of Public Health, Shandong University, Jinan, China; 2 Department of Epidemiology, Capital Institute of Pediatrics, Beijing, China; 3 Centre for Cellular and Molecular Biology, Council of Scientific and Industrial Research, Hyderabad, India; 4 Laboratory of Human Genetics, Beijing Hypertension League Institute, Beijing, China; University of Arkansas for Medical Sciences, United States of America

## Abstract

**Objective:**

Recent genome-wide association studies have identified many single nucleotide polymorphisms (SNPs) associated with body mass index (BMI)/generalized obesity. In this study, we aimed to examine the associations of identified SNPs with risk of central obesity in a child population from China.

**Methods:**

We genotyped 11 SNPs (*FTO* rs9939609, *MC4R* rs17782313, *GNPDA2* rs10938397, *BDNF* rs6265, *FAIM2* rs7138803, *NPC1* rs1805081, *SEC16B* rs10913469, *SH2B1* rs4788102, *PCSK1*rs6235, *KCTD15* rs29941, *BAT2* rs2844479) in the Chinese children (*N* = 3502, age range 6–18 years) from the Beijing Child and Adolescent Metabolic Syndrome (BCAMS). Based on the age- and sex- specific waist circumference (WC) standards generated in the BCAMS study, 1196 central obese cases and 2306 controls were identified.

**Results:**

Of 11 studied SNPs, four SNPs and genetic risk score (GRS) based on them were statistically significantly associated with central obesity by WC criteria (*FTO* rs9939609: OR = 1.29, 95%CI = 1.10–1.50, *p* = 0.001; *MC4R* rs17782313: OR = 1.27, 95%CI = 1.12–1.44, *p* = 1.32×10^−4^; *GNPDA2* rs10938397: OR = 1.22, 95%CI = 1.09–1.37, *p* = 4.09×10^−4^; *BDNF* rs6265: OR = 1.20, 95%CI = 1.08–1.34, *p* = 8.86×10^−4^; GRS: OR = 1.25, 95%CI 1.16–1.34, *p* = 2.58×10^−9^) after adjustment for sex, age, pubertal stage, physical activity and family history of obesity. Similar observations were made using weight-to-height ratio (WHtR) criterion. However, other SNPs were not associated with central obesity by WC as well as WHtR criterion.

**Conclusions:**

Our study replicates the statistically significant association of four SNPs (*FTO* rs9939609, *MC4R* rs17782313, *GNPDA2* rs10938397, *BDNF* rs6265) with risk of central obesity in the Chinese children.

## Introduction

Obesity is a serious public health issue worldwide including the developing countries like China [1], [2]. It is well established that childhood obesity is associated with many cardiovascular risk factors such as hypertension, dyslipidemia, metabolic syndrome and type 2 diabetes in children and in adults [3]. In addition, obesity at childhood may track into adulthood [4]. In majority of studies, obesity is usually defined according to body mass index (BMI) cut-offs. However, BMI can not provide information on the distribution of body fat [5]. Central obesity, as assessed by waist circumference (WC), is more strongly associated with the risk of hypertension, dyslipidaemia, type 2 diabetes, cardiovascular disease, cancer and all-cause mortality than generalized obesity as defined by BMI [6], [7]. Our recent study based on nearly 20,000 Chinese children also indicated that compared to individuals with generalized obesity, those with central obesity are more likely to have metabolic syndrome [8]. Thus, effective measures are required to prevent and control childhood central obesity in order to reduce the risk of chronic diseases in adult life.

Recently, genome-wide association studies (GWASs) have identified several BMI/generalized obesity susceptibility loci in the Europeans [9]–[18]. Subsequently, many studies have investigated the association of these loci with BMI in different ethnic populations. However, few studies have been conducted to examine the associations of these BMI-associated loci with central obesity. Because of the more important role of central obesity over generalized obesity, it is necessary to investigate whether BMI associated loci significantly predict central obesity so that effective strategies preventing and controlling childhood central obesity can be planned.

In our recent study on children and adolescents from the Beijing Child and Adolescent Metabolic Syndrome (BCAMS) study, we have confirmed the association of *FTO*, *MC4R*, *BDNF* and *GNPDA2* genes with BMI and generalized obesity, but have failed to replicate 7 other loci (*FAIM2, NPC1, SEC16B, SH2B1, PCSK1, KCTD15, BAT2*) [19]–[21]. In the current study, we aimed to examine the associations of these 11 GWAS-identified SNPs with central obesity and also to investigate the joint effect of these SNPs on central obesity among the Chinese children.

## Materials and Methods

### Study population

Subjects were recruited from the cross-sectional population-based BCAMS study, whose details are provided elsewhere [22]. The study included completion of a questionnaire, a medical examination, anthropometric measurements, and finger capillary blood tests from a representative sample of school-age children (n = 19593, age = 6–18 years, 50% boys) between April and October 2004. Anthropometric measurements included weight, height and WC. Pubertal development was assessed by Tanner stage of breast development (girls) and testicle volume (boys).

WC and waist-to-height ratio (WHtR, calculated as WC/height) were used as measures of central adiposity. We randomly recruited 3502 children from this large group of children, and used the 90th percentile values of WC for age and sex and WHtR cut-off of 0.5 to diagnose centrally obese children. Blood samples of all the children were collected by venipuncture.

### Ethics Statement

We obtained written informed consent from the parents/guardians of all participating children. The BCAMS study was approved by ethics committee of the Capital Institute of Pediatrics.

### Measurement of anthropometric parameters

All instruments were validated following the standard methods as per manufacturers' instructions and the investigators were properly trained based on the standard procedures recommended by World Health Organization [25]. WC was measured midway between the lowest rib and the superior border of the iliac crest at the end of normal expiration with an inelastic measuring tape to the nearest 0.1 cm. Height without shoes was measured using wall-mounted stadiometers to the nearest 0.1 cm. Body weight was measured with underwear and no shoes to the nearest 0.1 kg using beam scales with a maximum weight of 140 kg.

### Selection of SNPs and genotyping

We prepared a catalogue of SNPs that have been shown to be significantly associated with the risk of obesity by the GWAS [9]–[14]. To achieve a power of more than 0.75, we selected only SNPs in obesity-related genes with minor allele frequencies >0.10 in Chinese individuals in the HapMap database and 11 SNPs (rs9939609, rs17782313, rs10938397, rs6265, rs7138803, rs1805081, rs6235, rs29941, rs2844479, rs10913469, and rs4788102) fulfilled this criterion.

Genomic DNA was isolated from peripheral blood white cells using the salt fractionation method. SNPs were genotyped by TaqMan Allelic Discrimination Assays with the GeneAmp 7900 Sequence Detection System (Applied Biosystems, Foster City, CA) [26]. Genotyping call rates for all SNPs were greater than 99%. In order to validate the accuracy of genotyping, we repeated 70 samples randomly for each SNP and observed 100% concordance between the results of two tests.

### Statistical analysis

Continuous variables were expressed as means ± standard deviation, and differences between groups were assessed with Student's *t*-test. Categorical variables were represented as percentages and were tested by the χ^2^ test. Hardy-Weinberg equilibrium (HWE) was assessed using the χ^2^ test. The risk alleles of 11 SNPs were determined based upon the recent GWAS. The genetic risk score (GRS) was calculated as the sum of risk alleles of 4 SNPs (*FTO* rs9939609, *MC4R* rs17782313, *GNPDA2* rs10938397 and *BDNF* rs6265) that showed statistically significant association with central obesity even after Bonferroni correction for multiple testing. Association analysis of 11 SNPs and GRS with the risk of central obesity was performed using multivariate logistic regression model, assuming an additive model and with adjustment for sex, age, pubertal status, physical activity and family history of obesity. The Bonferroni correction was used to control for multiple testing (0.05/11 = 0.0045). Statistical analyses were performed with SPSS, version 13.0 (SPSS, Inc., Chicago, Illinois).

## Results

### Basic characteristics of the study population

A total of 1196 children were identified as centrally obese and 2306 as normal weight using the WC criterion and 1290 central obesity cases and 2212 controls using WHtR cut-offs [23], [24]. We noted significant differences in age, sex distribution, pubertal status, WC, WHtR, BMI, physical activity and family history of obesity between children with central obesity and control groups categorised either by WC (Table 1) or by WHtR (Table 2) (all *p*<0.01). All the SNPs were in HWE in controls (all *p*>0.05, Table 3).

**Table 1:** Basic characteristics of participants in the case-control study based on WC criteria.

	Central obesity	Controls	*p* value
*n*	1196	2306	
Sex (%male)	55.4	48.5	<0.001
Pubertal status(%)†			
I–IV	75.5	84.7	
V	24.5	15.3	<0.001
Age (years)	12.2±3.1	12.5±3.1	0.005
WC (cm)	84.6±10.3	66.0±9.3	<0.001
WHtR	0.55±0.04	0.44±0.04	<0.001
BMI (kg/m^2^)	26.5±3.7	19.5±3.6	<0.001
Physically active (%)‡	17.7	22.2	0.005
Family history of obesity (%)	30.4	18.6	<0.001

WC, waist circumference; WHtR, waist-to-height ratio; BMI, body mass index.

Data is presented as Mean±standard deviation unless indicated.

† Pubertal status was assessed by Tanner stage of breast development for girls and testicle volume for boys.

‡ Physically active subject was defined as participation in sports for at least 30 minutes per day and ≥5 days per week.

**Table 2:** Basic characteristics of participants in the case-control study based on WHtR criteria.

	Central obesity	Controls	*p* value
*n*	1290	2212	
Sex (%male)	66.9	41.5	<0.001
Pubertal status (%)†			
I–IV	78.0	83.6	
V	22.0	16.4	<0.001
Age (years)	12.2±2.9	12.5±3.1	0.004
WC (cm)	84.6±9.7	65.2±8.7	<0.001
WHtR	0.55±0.04	0.43±0.04	<0.001
BMI (kg/m^2^)	26.5±3.6	19.2±3.4	<0.001
Physically active (%)‡	17.6	22.4	0.002
Family history of obesity (%)	30.4	18.1	<0.001

WC, waist circumference; WHtR, waist-to-height ratio; BMI, body mass index.

Data is presented as Mean±standard deviation unless indicated.

† Pubertal status was assessed by Tanner stage of breast development for girls and testicle volume for boys.

‡ Physically active subject was defined as participation in sports for at least 30 minutes per day and ≥5 days per week.

**Table 3:** Minor allele frequency and Hardy-Weinberg equilibrium test in controls for each SNP.

SNP	MAF	*p* for HWE test	Effective sample size
**<90th percentile of WC**			
rs9939609	0.112	0.191	2305
rs17782313	0.219	0.974	2267
rs10938397	0.311	0.224	2264
rs6265	0.480	0.521	2269
rs7138803	0.282	0.461	2268
rs1805081	0.237	0.339	2262
rs6235	0.337	0.746	2306
rs29941	0.242	0.980	2306
rs2844479	0.440	0.823	2306
rs10913469	0.236	0.336	2306
rs4788102	0.159	0.507	2306
**<0.5 of WHtR**			
rs9939609	0.110	0.084	2212
rs17782313	0.220	0.687	2176
rs10938397	0.307	0.944	2171
rs6265	0.471	0.299	2177
rs7138803	0.285	0.439	2178
rs1805081	0.234	0.098	2170
rs6235	0.339	0.593	2212
rs29941	0.236	0.761	2212
rs2844479	0.442	0.607	2212
rs10913469	0.235	0.130	2212
rs4788102	0.158	0.972	2212

MAF, minor allele frequency; HWE, Hardy-Weinberg equilibrium; WC, waist circumference; WHtR, waist-to-height ratio.

### The associations of the 11 SNPs and GRS with WC, WHtR and central obesity

The minor allele frequencies of 11 SNPs showed in the current study were similar to those reported in the HapMap Han Chinese (Table 3). After Bonferroni correction for multiple comparisons, only three SNPs (*FTO* rs9939609, *MC4R* rs17782313, *GNPDA2* rs10938397) and GRS were statistically significantly associated with both WC and WHtR (3.09×10^−12^<*p*<0.001) (Table S1). Four SNPs (*FTO* rs9939609, *MC4R* rs17782313, *GNPDA2* rs10938397, *BDNF* rs6265) and GRS based on these four SNPs were significantly associated with central obesity by WC criteria (*FTO* rs9939609: OR = 1.29, 95%CI = 1.10–1.50, *p* = 0.001; *MC4R* rs17782313: OR = 1.27, 95%CI = 1.12–1.44, *p* = 1.32×10^−4^; *GNPDA2* rs10938397: OR = 1.22, 95%CI = 1.09–1.37, *p* = 4.09×10^−4^; *BDNF* rs6265: OR = 1.20, 95%CI = 1.08–1.34, *p* = 8.86×10^−4^; GRS: OR = 1.25, 95%CI = 1.16–1.34, *p* = 2.58×10^−9^) after adjustment for sex, age, pubertal stage, physical activity and family history of obesity and correction for multiple testing (Table 4). We observed similar results for 11 SNPs with central obesity by WHtR criteria (Table 4).

**Table 4:** Associations of 11 variants with central obesity in the Chinese children.

SNP	Gene	Risk/Non-risk allele	OR^a^	95% CI^a^	*p* value^a^	
**≥90th percentile of WC**						
rs9939609	*FTO*	A/T	1.29	1.10	1.50	**0.001**
rs17782313	*MC4R*	C/T	1.27	1.12	1.44	**1.32×10^−4^**
rs10938397	*GNPDA2*	G/A	1.22	1.09	1.37	**4.09×10^−4^**
rs6265	*BDNF*	G/A	1.20	1.08	1.34	**8.86×10^−4^**
rs7138803	*FAIM2*	A/G	1.13	1.01	1.27	0.031
rs1805081	*NPC1*	A/G	1.08	0.95	1.22	0.240
rs6235	*PCSK1*	C/G	0.95	0.85	1.06	0.357
rs29941	*KCTD15*	C/T	1.04	0.91	1.17	0.584
rs2844479	*BAT2*	T/G	1.02	0.92	1.14	0.653
rs10913469	*SEC16B*	C/T	1.04	0.92	1.18	0.547
rs4788102	*SH2B1*	A/G	1.10	0.96	1.27	0.168
Genetic risk score			1.25	1.16	1.34	**2.58×10^−9^**
≥0.5 of WHtR						
rs9939609	*FTO*	A/T	1.33	1.14	1.56	**3.56×10^−4^**
rs17782313	*MC4R*	C/T	1.25	1.10	1.42	**4.71×10^−4^**
rs10938397	*GNPDA2*	G/A	1.25	1.11	1.39	**1.32×10^−4^**
rs6265	*BDNF*	G/A	1.06	0.95	1.18	0.310
rs7138803	*FAIM2*	A/G	1.08	0.96	1.22	0.175
rs1805081	*NPC1*	A/G	1.05	0.93	1.19	0.421
rs6235	*PCSK1*	C/G	0.91	0.82	1.03	0.128
rs29941	*KCTD15*	C/T	1.12	0.99	1.27	0.073
rs2844479	*BAT2*	T/G	1.00	0.90	1.11	0.961
rs10913469	*SEC16B*	C/T	1.06	0.94	1.20	0.364
rs4788102	*SH2B1*	A/G	1.11	0.97	1.29	0.138
Genetic risk score			1.26	1.17	1.36	**6.60×10^−10^**

a Adjusted for sex, age, pubertal status, physical activity and family history of obesity; SNPs reaching Bonferroni corrected p value of 0.0045 are represented in bold.

## Discussion

In the present study, we investigated associations of 11 BMI-associated loci with risk of central obesity in the Chinese children. The results indicated that four SNPs (*FTO* rs9939609, *MC4R* rs17782313, *GNPDA2* rs10938397, *BDNF* rs6265) and GRS calculated from these 4 SNPs significantly predicted the risk of central obesity. However, we failed to observe the associations of seven other BMI-associated loci with central obesity.

Frayling et al. firstly reported that *FTO* gene was significantly associated with generalized obesity in European adults and children [9]. Since then, many studies have examined the association among different ethnic populations [27]–[29]. Although contradictory results were reported, three recent meta-analyses have confirmed the significant association of *FTO* gene with BMI and generalized obesity [27]–[29]. Two other obesity associated genes, *MC4R* and *GNPDA2* were also identified by GWAS in Europeans and replicated in other studies [10], [12]. A 12-year longitudinal study showed that *FTO* rs8050136 and *GNPDA2* rs10938397 SNPs, rather than *MC4R* rs17782313, predicted persistent central obesity in the Chinese adults [30]. However, it should be noted that only 354 subjects with central obesity were recruited in the abovementioned study and hence was underpowered to detect the association. Another study from Japan also demonstrated significant association between *FTO* rs1558902 SNP and central obesity in adults [31]. However, to our knowledge, there is no published study on the association analysis of these BMI-associated loci with central obesity in pediatric population. Our study presents the new evidence that *FTO* rs9939609, *MC4R* rs17782313, *GNPDA2* rs10938397 and *BDNF* rs6265 are statistically significantly associated with risk of central obesity in the Chinese children.

The mechanisms of how BMI associated SNPs influence central obesity are unclear. FTO and MC4R proteins are highly expressed in hypothalamus, which regulates the energy balance [12]. Many studies have indicated that *FTO* and *MC4R* SNPs influence energy-dense food intake rather than regulation of energy expenditure [32], [33]. Other studies have suggested that *FTO* and *MC4R* SNPs are associated with metabolic traits (higher fasting insulin, glucose, triglycerides and lower HDL cholesterol) that are mediated through BMI. Future studies focusing on these phenotypes might help in clarifying the mechanisms through which these loci and central obesity are related.

The joint effect of four strongly associated SNPs (*FTO* rs9939609, *MC4R* rs17782313, *GNPDA2* rs10938397, *BDNF* rs6265) on the risk of central obesity was further investigated. It is worth mentioning that the age- and sex-adjusted OR of GRS for central obesity was only 1.25, which points to their limited predictive effect on risk of central obesity in the Chinese children. However, the genetic information may be useful to identify high-risk children who may need early interventions such as lifestyle modifications and individualized management strategies, in order to reduce the incidence of obesity related diseases.

Our study has two strengths; firstly, we achieved sufficient power (>0.75) for each SNP and secondly, the findings are novel for several SNPs since very few studies have focused on the associations between BMI associated SNPs and central obesity. However, few limitations should also be noted. First, the case-control design does not allow interpreting causality of the associations. Second, recent GWASs have identified a large number of SNPs to be associated with BMI/generalized obesity [15]–[18] and hence, future studies would need to take more SNPs into account, while conducting such association analyses. Finally, the findings of our study may need to be replicated in other pediatric cohort.

In conclusion, our study confirmed the significant association of four SNPs (*FTO* rs9939609, *MC4R* rs17782313, *GNPDA2* rs10938397, *BDNF* rs6265) with risk of central obesity in the Chinese children. Large-scale longitudinal studies with consideration of gene-gene and gene-environment interactions should be conducted to investigate the causality of these associations in future.

## Supporting Information

Table S1 **Associations of 11 variants with waist circumference (WC) and weight to height ratio (WHtR) in Chinese children.**
(DOC)
