# Supplementary material for: Study of 11 BMI-Associated Loci Identified in GWAS for Associations with Central Obesity in the Chinese Children
Source: PLoS One. 2013 Feb 12;8(2):e56472. doi: 10.1371/journal.pone.0056472 (PMC3570414; doi:10.1371/journal.pone.0056472)
Supplement: Table S1 — Associations of 11 variants with waist circumference (WC) and weight to height ratio (WHtR) in Chinese children. (DOC) [file pone.0056472.s001.doc]

| Table S1: Associations of 11 variants with waist circumference (WC) and weight to height ratio (WHtR) in Chinese children | | | | | | |
| --- | --- | --- | --- | --- | --- | --- |
| **SNP** | **Risk/**  **Non-risk allele** | **Gene** | ***β*^a^** | **95% CI^a^** | | ***p* value ^a^** |
| **WC, cm** |  |  |  |  |  |  |
| rs9939609 | A/T | *FTO* | 1.73 | 0.91 | 2.55 | **3.59×10^-5^** |
| rs17782313 | C/T | *MC4R* | 1.57 | 0.93 | 2.22 | **1.94×10^-6^** |
| rs10938397 | G/A | *GNPDA2* | 0.97 | 0.39 | 1.55 | **0.001** |
| rs6265 | G/A | *BDNF* | 0.40 | -0.15 | 0.95 | 0.150 |
| rs7138803 | A/G | *FAIM2* | 0.69 | 0.09 | 1.28 | 0.024 |
| rs1805081 | A/G | *NPC1* | 0.22 | -0.42 | 0.86 | 0.496 |
| rs6235 | C/G | *PCSK1* | -0.16 | -0.74 | 0.42 | 0.590 |
| rs29941 | C/T | *KCTD15* | 0.62 | -0.03 | 1.26 | 0.060 |
| rs2844479 | T/G | *BAT2* | 0.09 | -0.46 | 0.64 | 0.737 |
| rs10913469 | C/T | *SEC16B* | 0.66 | 0.02 | 1.30 | 0.043 |
| rs4788102 | A/G | *SH2B1* | 0.46 | -0.27 | 1.20 | 0.217 |
| Genetic risk score |  |  | 1.34 | 0.96 | 1.73 | **8.16×10^-12^** |
| **WHtR** |  |  |  |  |  |  |
| rs9939609 | A/T | *FTO* | 0.011 | 0.006 | 0.016 | **1.26×10^-5^** |
| rs17782313 | C/T | *MC4R* | 0.009 | 0.005 | 0.013 | **4.59×10^-6^** |
| rs10938397 | G/A | *GNPDA2* | 0.006 | 0.003 | 0.009 | **0.001** |
| rs6265 | G/A | *BDNF* | 0.002 | -0.001 | 0.006 | 0.154 |
| rs7138803 | A/G | *FAIM2* | 0.004 | 0.001 | 0.008 | 0.021 |
| rs1805081 | A/G | *NPC1* | 0.002 | -0.002 | 0.006 | 0.288 |
| rs6235 | C/G | *PCSK1* | -0.002 | -0.005 | 0.002 | 0.372 |
| rs29941 | C/T | *KCTD15* | 0.003 | 0.000 | 0.007 | 0.076 |
| rs2844479 | T/G | *BAT2* | 0.001 | -0.002 | 0.005 | 0.403 |
| rs10913469 | C/T | *SEC16B* | 0.003 | -0.001 | 0.007 | 0.112 |
| rs4788102 | A/G | *SH2B1* | 0.003 | -0.002 | 0.007 | 0.246 |
| Genetic risk score |  |  | 0.008 | 0.006 | 0.010 | **3.09×10^-12^** |

^a^Adjusted for sex, age, pubertal status, physical activity and family history of obesity; SNPs reaching Bonferroni corrected *p* value of 0.0045 are represented in bold.
